# Exploring transvaginal sonographic characteristics of the levator ani muscle in women with postpartum pelvic floor myofascial pain

**DOI:** 10.1186/s12905-024-03052-9

**Published:** 2024-04-18

**Authors:** Juntong Ye, Hui Fei, Jingran Du, Yun Liu, Juan He, Mengxiong Li, Yunxia He, Pinyu Ren, Juanhua Li, Yang Xu, Jing Li, Pu Wang, Xinling Zhang, Tian Li

**Affiliations:** 1 The Pelvic Floor Disorder Center, Obstetrics and Gynecology Department, The Seventh Affiliated Hospital, Sun Yat-sen University, Guangdong Province, Shenzhen, China; 2 Department of Ultrasound, The Third Affiliated Hospital of Sun Yat-Sen University, Guangdong Province, Guangzhou, China

**Keywords:** Pelvic floor myofascial pain, Injury of the levator ani muscles, Transvaginal ultrasound, Postpartum, Localization of trigger points

## Abstract

**Background:**

Pelvic floor myofascial pain is one of the pelvic floor dysfunction diseases disturbing women after delivery. There is a lack of objective standardization for the diagnosis of pelvic floor myofascial pain due to the various symptoms and the dependence on the palpating evaluation. Ultrasound imaging has the advantages of safety, simplicity, economy and high resolution, which makes it an ideal tool for the assistant diagnosis of pelvic floor myofascial pain and evaluation after treatment.

**Methods:**

This is a retrospective case-control study including women accepting evaluation of pelvic floor function at 6 weeks to 1 year postpartum. They were divided into pelvic floor myofascial pain group and normal control group. A BCL 10–5 biplane transducer was applied to observed their puborectalis. The length, minimum width, area, deficiency, deficiency length, deficiency width, deficiency area, rate of deficiency area, local thickening,angle between the tendinous arch of levator ani muscle and puborectalis of corresponding puborectalis in different groups were observed and measured.

**Results:**

A total of 220 postpartum women participated in the study, with 77 in the pelvic floor myofascial pain group and 143 in the normal control group. The Intraclass correlation coefficient value was over 0.750, and Kappa ranged from 0.600 to 0.800. puborectalis deficiency (adjusted odds ratio = 11.625, 95% confidence interval = 4.557–29.658) and focal thickening (adjusted odds ratio = 16.891, 95% confidence interval = 1.819–156.805) were significantly associated with higher odds of having postpartum pelvic floor myofascial pain. Grayscale or the angle between the arch tendineus levator ani and puborectalis measurements on the pain side tended to be smaller than on the non-pain side in patients with unilateral puborectalis or iliococcygeus pain (*P* < 0.05).

**Conclusions:**

This study demonstrated that transvaginal ultrasound was a potentially efficient technique for evaluating postpartum pelvic floor myofascial pain due to its ability to assess various sonographic characteristics of the levator ani muscles.

**Supplementary Information:**

The online version contains supplementary material available at 10.1186/s12905-024-03052-9.

## Introduction

Pelvic floor myofascial pain (PFMP) refers to pain originating from the pelvic floor muscles, including levator ani muscle (LAM) and obturator internus (OI) [1]. PFMP is commonly found in women with pelvic floor dysfunctions, particularly those experiencing chronic pelvic pain [2]. Previous research found that pregnant women with pelvic girdle pain often have concomitant pelvic floor myofascial pain, which may persist even after delivery [3]. It has been reported that approximately 50% of women within the first year postpartum may develop PFMP due to injury to the levator ani muscles during pregnancy and vaginal delivery [3]. Patients with PFMP present pelvic floor pain associated with myofascial tension or spasm, accompanied by highly sensitive trigger points [4]. However, some patients may not exhibit pain symptoms but can still have moderate to severe pain upon palpating their pelvic floor muscles [4]. Unfortunately, there is currently no standardized palpation process for evaluating PFMP. Meister et al. [4] summarized a “clock face” palpating method and recommended self-reported scales to assess the level of pain upon palpation based on the limited consensus reported in 55 literatures. However, variations in palpation force and the subjectivity of self-reported pain may result in inconsistent assessments of PFMP and its severity. Therefore, there is a need for developing imaging methods, with more objective measurements, to improve the reliability and sensitivity for evaluating PFMP.

Ultrasound imaging has been utilized for evaluating pelvic floor dysfunction for many years, offering various advantages such as safety, simplicity, affordability, high resolution, absence of radiation, and dynamic observation [5]. Recent studies have validated the reliability of transvaginal ultrasound in assessing the structure of the levator ani muscles through systematic autopsies. The high resolution and detailed identification ability of the levator ani muscle with ultrasound enable a more precise assessment of the structure and injuries of these muscles [6, 7]. Additionally, ultrasound is widely accepted for evaluating myofascial pain, with specific characteristics such as hypoechoic regions (darker grayscale) at the trigger points and increased stiffness in elastography [8]. Ultrasound serves not only to detect myofascial trigger points in myofascial pain syndrome but also enhances the accuracy of trigger point localization, thereby improving the effectiveness and safety of intervention therapy. A systematic review by Dion et al. [9] highlighted the use of ultrasound-guided intervention therapy for myofascial pain, demonstrating superior pain improvement compared to blind intervention groups, with minimal self-limited adverse events in all studies. While various studies have evaluated the application of ultrasound in diagnosing and treating myofascial pain syndrome, no reports have been made on its application in pelvic floor myofascial pain. Given the high prevalence of postpartum pelvic floor myofascial pain and the advantages of transvaginal ultrasound in observing the levator ani muscle, this study utilized transvaginal ultrasound to investigate the levator ani muscle in women within 1 year after delivery and with pelvic floor myofascial pain. The study aimed to compare the characteristics and parameter changes of the levator ani muscle between normal postpartum women and those with pelvic floor myofascial pain, providing imaging evidence for the future diagnosis and treatment of postpartum pelvic floor myofascial pain.

## Method

### Participants

A total of 290 postpartum women who underwent pelvic floor function examination at the Pelvic Floor Center of the Seventh Affiliated Hospital of Sun Yat-sen University from April 2022 to November 2022 were included in the study. Inclusion criteria were women aged 18–45 years, more than 6 weeks, and less than 12 months postpartum. Exclusion criteria included inability to cooperate with vaginal palpatiodand transvaginal pelvic floor ultrasound examination, stage II or above pelvic floor organ prolapse [10], moderate or above urinary incontinence(Ingelman-Sundberg classification), vaginal bleeding, history of pelvic floor-related surgery, urinary tract infection, endometriosis, inflammation of the reproductive system, pelvic trauma and other specific diseases inducing pain, and history of the pelvic malignant tumor.

### Diagnosis of PFMP

PFMP was diagnosed as pain in the pelvic floor, which may manifest as symptoms of overactive bladder, constipation or dyspareunia, or no spontaneous pain, only pain during palpation of the levator ani muscle and obturator internus muscle, often accompanied by a highly sensitive trigger point [4]. Two attending obstetricians and gynecologists with more than 5 years of experience in the diagnosis and treatment of pelvic floor dysfunction and who underwent unified training conducted palpation using the “clock face” method. The participants lay supine in a lithotomy position, and the examiner used their index finger to palpate and locate the pelvic floor muscles from superficial to deep layers following a 0–12 o’clock orientation. During palpation, the participants were instructed to report the degree of pain using Visual Analogue Scale (VAS) [11]. The pain scale ranged from 0 to 10 cm, with 0 cm anchoring at “no pain” and 10 cm at “worst pain ever experienced.”

### Ultrasound imaging

**Fig. 1:**
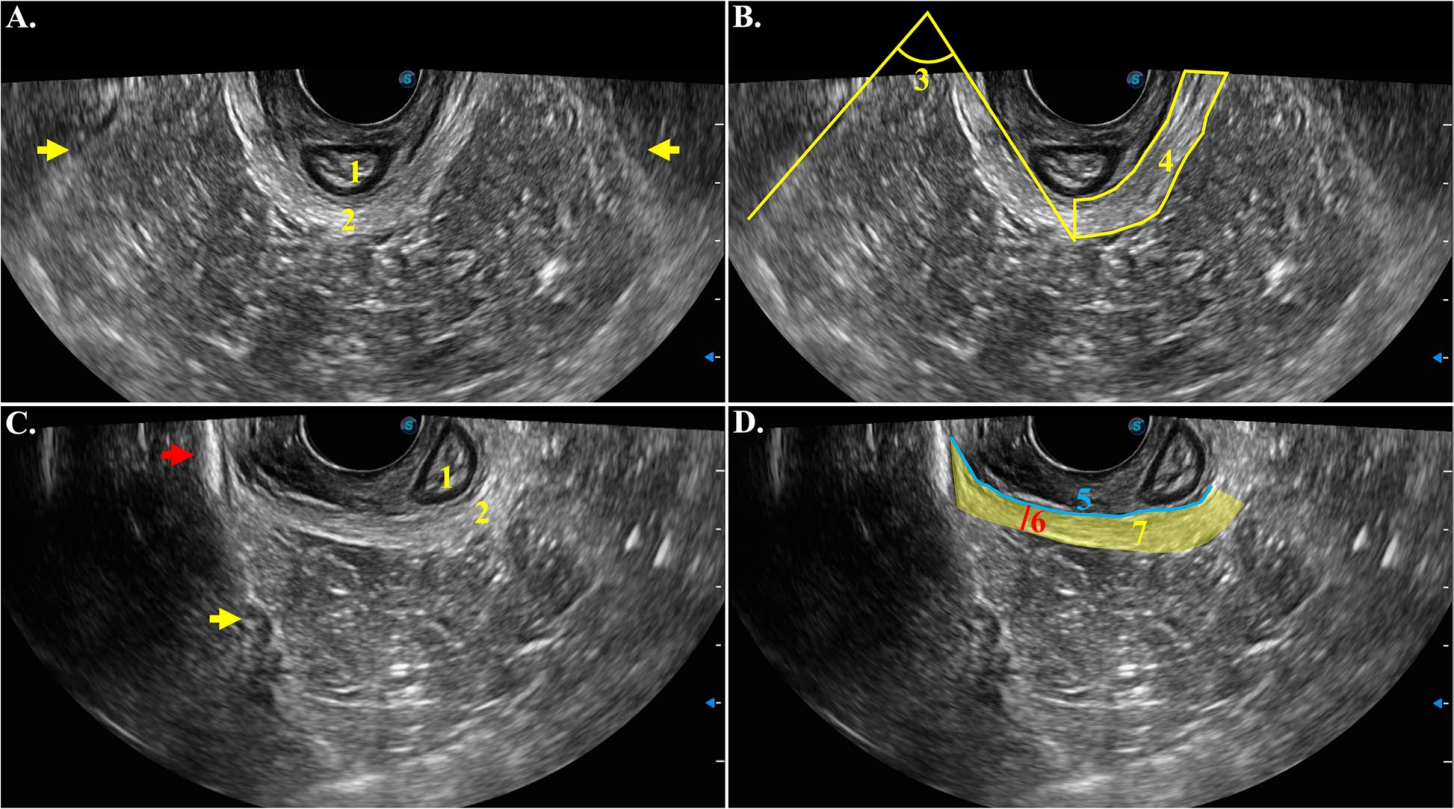
Transvaginal ultrasound showing the axial plane of the puborectalis (PR) in a normal woman. Yellow arrows (**A**, **C**) indicate the arch tendinous levator ani (ATLA). The red arrow (**C**) indicates the pubis branch. 1, anal canal; 2, PR; 3, angle between the ATLA and PR (AAP), 74.386°; 4, measurement region of grayscale of the left PR, indicted by yellow frame. The grayscale is 142.152; 5, length of the right PR, 5.033 cm; 6, minimum width of the right PR, 0.416 cm; 7, area of the right PR, indicated by yellow shade, 2.994 cm^2^

An ultrasound imaging unit (X5; SonoScape Medical Corp., Shenzhen, China) with an 8.5–11.2 MHz biplane vaginal transducer which had a maximum scanning angle of 200°, to evaluate the levator ani muscles in postpartum women. The puborectalis (PR) and iliococcygeus (IC) are the dominant muscles of the levator ani muscle. However, due to limitations in imaging, the IC muscle could not be satisfactorily and entirely displayed [12]. Therefore, the evaluation of levator ani muscle in this study mainly focused on the PR muscle. B-mode grayscale imaging with a depth of 11 was used. The imaging procedure was performed by a gynecologist who had received professional training and had more than 5 years of experience in the diagnosis and treatment of pelvic floor dysfunction, as well as more than 3 years of experience in pelvic floor ultrasonography. The participant lied in supine with the knees flexed to the maximum. The vaginal transducer was placed in the vagina and positioned to ensure that the anal canal, PR muscle, and arch tendineus levator ani (ATLA) were displayed symmetrically in the axial plane. A U-shaped PR muscle was then visualized and captured. Subsequently, the transducer was rotated to the left and right to visualize the attachment positions of the PR muscle to the pubic ramus on both sides in order to obtain clear Images of the PR muscle and its attachments (Fig. 1).

### Observational or measured indicators of the images

**Fig. 2:**
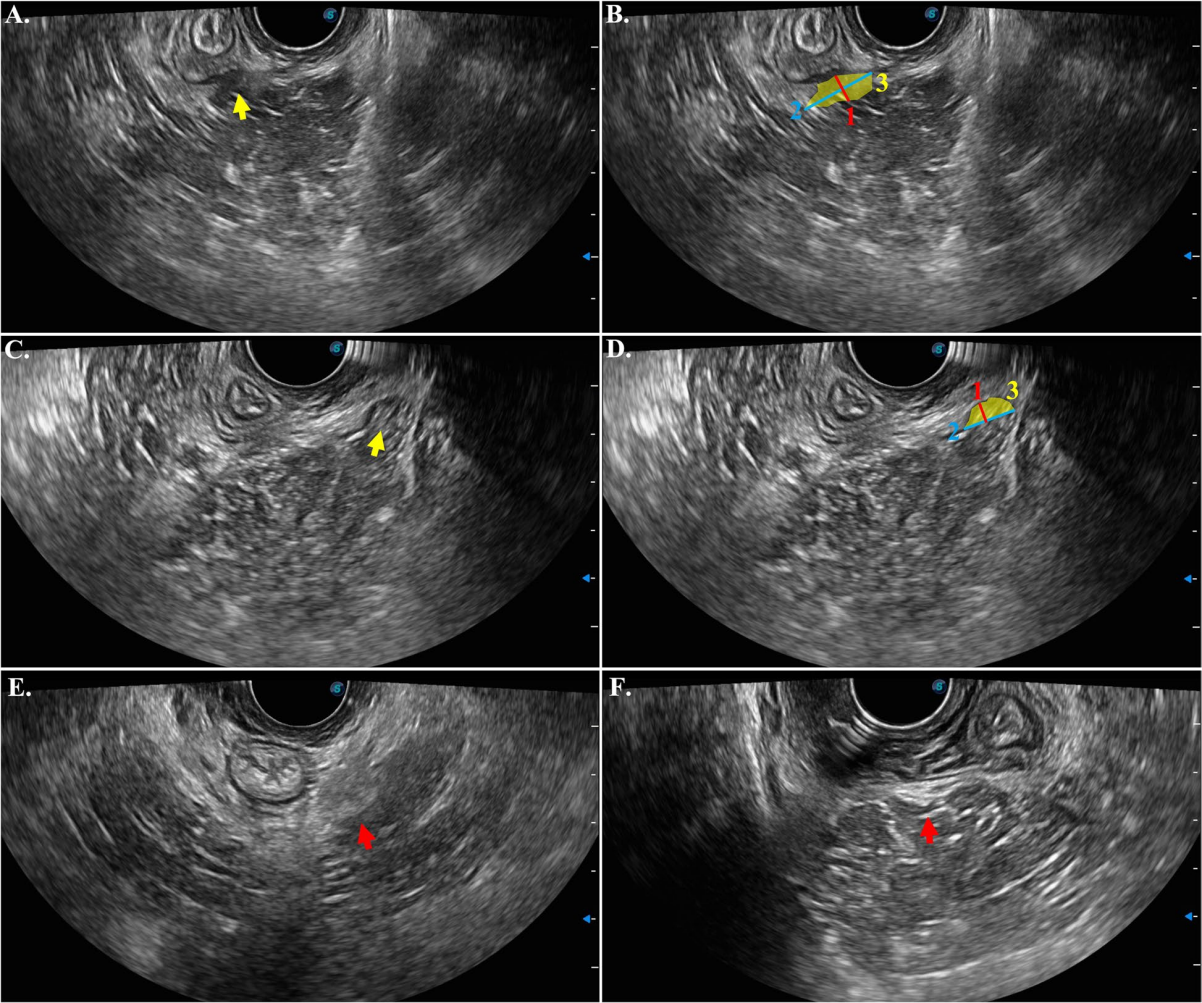
Deficiency (**A-D**) and focal thickening (**E**, **F**) of the puborectalis (PR) in transvaginal ultrasound. Images were from 4 different women with PFMP, and their symptoms of them were left PR pain (**A**, **E**), right PR pain (**F**), and left iliococcygeus pain (**C**). Yellow arrows (**A**, **C**) indicate deficiency. **B**, **D**. Measurements of deficiency: 1. width of deficiency; 2. length of deficiency; 3. area of deficiency, indicated by yellow shade. Red arrows (**E**, **F**) indicate focal thickening

Observational and measurement indicators were used to evaluate the ultrasound images, as described below (Figs. 1 and 2). The measurement was performed using Image J 2.1.0 by two physiotherapists with 3 and 5 years of experience in the diagnosis and treatment of pelvic floor muscle dysfunction.

A Grayscale: The mean grayscale value was measured from the upper edge of the left puborectalis (LPR)/ right puborectalis (RPR) to the 6 o’clock position in the axial plane. Three measurements were taken on each side, and the average of the three values was recorded.
B Angle between the arch tendineus levator ani (ATLA) and puborectalis muscle (AAP): The angle formed between the ATLA and the midpoint of the puborectalis muscle on the left and right sides was measured.
C Length: The length of the inner border of the attachment of the unilateral puborectalis muscle to the pubic ramus to the 6 o’clock position on LPR/RPR was measured.
D *minimum* width: the width of the narrowest position on LPR/ RPR was measured.
E Area: the area from the LPR/RPR attachment to the 6 o’clock position was measured.
F Deficiency: Low echo changes in LPR/RPR, such as avulsion, inflammation, and partial defect, were measured. The length of the long and short axis as well as the area of the deficiency portion were measured. The transducer was moved up and down to evaluate the deficiency of the entire PR during observation.
G Focal thickening: The thickened nodules in LPR/RPR were evaluated by moving the transducer up and down to assess the focal thickening of the entire puborectalis muscle.

Both the front and lateral views were examined to exclude the artifacts caused by the volume effect when evaluating deficiency or focal thickening.

### Statistical analysis

Quantitative data with normal distribution are presented as mean ± standard deviation (SD), and non-normal distribution is presented as median (interquartile range). Qualitative data are presented as frequency. SPSS 25.0 (IBM, Armonk, NY, USA) was used for statistical analysis. Consistency analysis between the two measures was evaluated using the Intraclass correlation coefficient (ICC). ICC values less than 0.40 were considered poor consistency, 0.40–0.59 as general, 0.60–0.74 as moderate, and 0.75–1 as excellent [13]. Categorical or graded data were evaluated using Cohen’s Kappa coefficient. Kappa values less than 0.2 represented poor consistency, 0.2–0.4 represented fair, 0.4–0.6 represented moderate, 0.6–0.8 represented good, and 0.8–1 represented excellent [7]. Difference analysis was based on the measurement by physicians with 5 years of work experience. The Independent sample T-test was used for data with normal distribution, the Mann-Whitney U test for data with non-normal distribution and rank variables, and the chi-square test was used for enumeration data. Fisher’s exact probability method was used if the theoretical frequency was less than 5. Spearman’s correlation coefficient was used to evaluate the correlation between the measurement events and the Visual Analog Scale (VAS) score. Paired data were analyzed using the Wilcoxon signed-rank test and McNemar’s test. Logistic regression was employed to examine the relationship between the measurement events and PFMP while adjusting for relevant factors identified through univariate analysis and reported in previous literature, including age, BMI, education, postpartum days, mode of delivery (cesarean or spontaneous), operative vaginal delivery, parity, birth weight, episiotomy, laceration, lactation and urinary incontinence [3, 14–18]. A significance level of *P* < 0.05 was used to determine statistical significance.

## Result

**Fig. 3:**
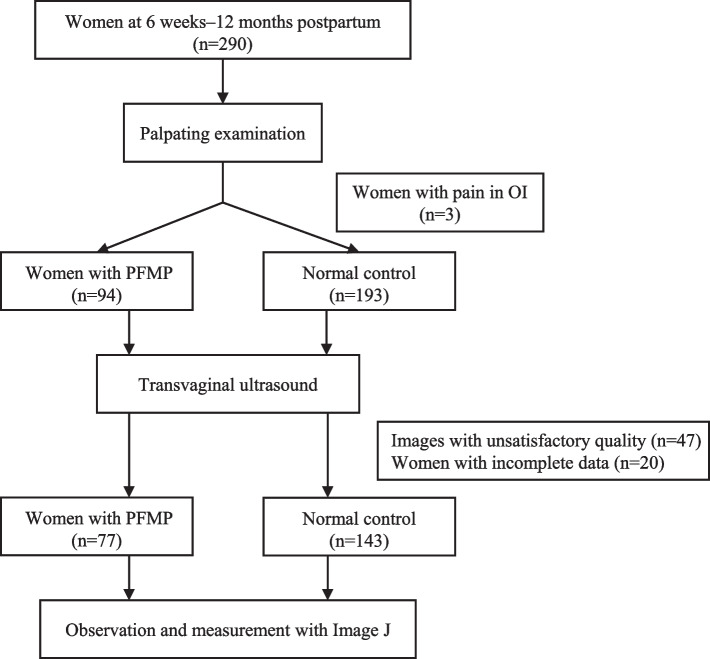
Flow chart of the study. PFMP, pelvic floor myofascial pain; OI, obturator internus. Women with pain in the OI were excluded because of the very small sample size. Women with unsatisfied images or incomplete data were excluded

Out of the 290 participants, there were 193 normal controls and 97 women with PFMP. Among women with PFMP, 50 cases (53.2%) reported pain in the puborectalis (PR), 47 cases (50.0%) reported pain in the iliococcygeus (IC), and 3 cases (3.2%) reported pain in the obturator internus (OI). Women with pain in the OI were excluded from the final analysis due to the small sample size. After excluding cases with pain in the OI, 240 (83.6%) of 287 postpartum women successfully obtained good-quality ultrasound images, while 47 with unsatisfactory-quality ultrasound images, and 20 with incomplete data were excluded. The remaining 220 participants included 77 women in the PFMP group and 143 women in the normal control group (Fig. 3), with an average age of 30.95 ± 4.28 years. Among women with PFMP, 42 (54.5%) reported pain in the left puborectalis (LPR), 20 (26.0%) reported pain in the right puborectalis (RPR), 27 (35.1%) reported pain in the left iliococcygeus (LIC), and 35 (45.5%) reported pain in the right iliococcygeus (RIC). The baseline characteristics of the PFMP group and the control group are shown in Table 1. The proportion of postpartum women in the PFMP group who were breastfeeding was lower than that in the control group (84.4% vs. 94.4%, *P < 0.05*). The number of vaginal births in the PFMP group (median 1, IQR 1–2) was higher than that in the control group (median 1, IQR 0–2; *P* < 0.05]. The proportion of urinary incontinence (mild) in the PFMP group was higher than that in the control group (32.5% vs. 16.1%, *P* < 0.01).

**Table 1:** The baseline of the women with PFMP and the normal control after delivery

Baseline	PFMP(*n* = 77)	Normal control(*n* = 143)	Z/t/𝜒2	*P* value
Age	30.740 ± 4.706	31.070 ± 4.041	0.544	0.587
Education			0.903	0.342
High school	45 (58.4%)	74 (51.7%)		
College	32 (41.6%)	69 (48.3%)		
Height	158.721 ± 4.272	160.017 ± 4.935	1.946	0.053
Weight	55.196 ± 6.619	56.508 ± 6.767	1.382	0.168
BMI	21.898 ± 2.481	22.051 ± 2.508	0.434	0.677
Lactation	65 (84.4%)	135 (94.4%)	6.044	0.014
Gestational week	39.10 (38.20, 40.0)	39.00 (38.50, 39.60)	−0.033	0.973
Mode of delivery			2.181	0.14
Vaginal birth	60 (77.9%)	98 (68.5%)		
C-section	17 (22.1%)	45 (31.5%)		
Postpartum days	56.00 (49.00, 68.50)	53.00 (47.00, 66.00)	0.863	0.388
Gravidity	2.00 (1.00, 3.00)	2.00 (1.00, 2.00)	0.872	0.383
Parity	1.00 (1.00, 2.00)	1.00 (1.00, 2.00)	0.463	0.643
Number of vaginal births	1.00 (1.00, 2.00)	1.00 (0.00, 2.00)	1.999	0.046
Number of C-sections	0.00 (0.00,0.00)	0.00 (0.00, 1.00)	−1.833	0.067
Episiotomy	16 (20.8%)	26 (18.2%)	0.219	0.64
Laceration	34 (44.2%)	57 (39.9%)	0.381	537
Operative delivery	3 (3.9%)	3 (2.1%)	0.61	0.435
Birth weight	3.090 (2.845, 3.425)	3.200 (2.950, 3.500)	−1.661	0.097
Urinary incontinence	25 (32.5%)	23 (16.1%)	7.876	0.005
POP-Q				
Anterior vaginal wall			0.004	0.951
0	1 (1.3%)	2 (1.4%)		
I	76 (98.7%)	141 (98.6%)		
Uterus/fornix			0.037	0.848
0	27 (35.1%)	52 (36.4%)		
I	50 (64.9%)	91 (63.6%)		
Posterior vaginal wall				
0	13 (16.9%)	34 (23.8%)	1.416	0.234
I	64 (83.1%)	109 (76.2%)		
Strength of PFM				
Type I muscle			1.093	0.579
I	52 (67.5%)	94 (65.7%)		
II	21 (27.3%)	36 (25.2%)		
III	4 (5.2%)	13 (9.1%)		
Type II muscle			2.209	0.53
I	21 (27.3%)	32 (22.4%)		
II	30 (39.0%)	64 (44.8%)		
III	20 (26.0%)	41 (28.7%)		
IV	6 (7.8%)	6 (4.2%)		

*PFMP* pelvic floor myofascial pain, *SEMG* surface electromyography, *POP-Q* Pelvic Organ Prolapse Quantification, *PFM* pelvic floor muscle

Continuous variables with a normal distribution are presented as mean ± SD (standard deviation) and compared using independent samples t-test

Continuous variables with a non-normal distribution are presented as median (interquartile range) and compared using the Mann-Whitney U test

Categorical variables are presented as numbers (percentages) and compared using the Chi-square test

Significant differences in lactation, number of vaginal births, and urinary incontinence were found between the women with PFMP and the normal control group

The evaluation of the interrater reliability showed that the reliability of each indicator was excellent (ICC > 0.750) except for the grayscale of RPR (moderate, ICC = 0.742). The reliability of deficiency and focal thickening in LPR and RPR was good (Kappa = 0.600–0.800) (Table A.1-A.2).

### Comparison between women with PR pain and the control group

In comparison to the control group, women with LPR pain showed higher frequencies of deficiency and focal thickening in the LPR (52.4% vs. 5.6%, *P* < 0.001; 19.0% vs. 0, *P* < 0.001). Similarly, women with RPR pain had higher frequencies of deficiency and focal thickening in the RPR compared to the control group (45.0% vs. 1.4%, *P* < 0.001; 10.0% vs. 0.7% *P* < 0.05) (Tables 2 and 3). The length, minimum width, area, grayscale, and AAP in the LPR/RPR were also compared between women with LPR/RPR pain and the control group. The LPR length was found to be longer in patients with LPR pain and the control group (median 5.398, IQR 5.174–5.866 vs. median 5.237, IQR 4.939–5.545, *P* < 0.05). However, no correlation was found between the measurement indicators and the VAS score of both LPR and RPR pain (*P* > 0.05).

**Table 2:** Observational and measured characteristics in the LPR of women with LPR/ LIC pain and normal control

Characteristic	Normal Control (*n* = 143)	LPR pain (*n* = 42)	*P* value^†^	LIC pain (*n* = 27)	*P* value^‡^
Deficiency	8 (5.6%)	22 (52.4%)	< 0.001	8 (29.6%)	< 0.001
Focal thickening	0	8 (19.0%)	< 0.001	0	–
Length (cm)	5.237 (4.939, 5.545)	5.398 (5.174, 5.866)	0.018	5.327 (4.851, 5.946)	0.333
Minimum width (cm)	0.442 (0.374, 0.517)	0.426 (0.380, 0.554)	0.784	0.410 (0.357, 0.489)	0.363
Area (cm^2^)	3.675 (3.138, 4.210)	3.620 (3.350, 4.140)	0.671	3.394 (3.085, 4.234)	0.698
Grayscale	127.979 ± 15.104	124.974 ± 13.084	0.245	122.008 ± 13.340	0.057
AAP (°)	79.142 (72.062, 85.258)	80.213 (70.865, 86.123)	0.741	77.730 ± 10.420	0.692

*LPR* left puborectalis, *LIC* left iliococcygeus, AAP, the angle between the arch tendineus levator ani and puborectalis

^†^Comparisons between the characteristics in the LPR of women with LPR pain and normal controls

^‡^Comparisons between the characteristics in the LPR of women with LIC pain and normal controls

**Table 3:** Observational and measured characteristics in the RPR of women with RPR/RIC pain and normal control

Characteristic	Normal control (*n* = 143)	RPR pain (*n* = 20)	*P* value^†^	RIC pain (*n* = 35)	*P* value^‡^
Deficiency	2 (1.4%)	9 (45.0%)	< 0.001	3 (8.6%)	0.053
Focal thickening	1 (0.7%)	2 (10.0%)	0.040	0	1
Length (cm)	5.328 ± 0.572	5.463 ± 0.577	0.326	5.526 ± 0.590	0.07
Minimum width (cm)	0.469 ± 0.122	0.471 ± 0.142	0.944	0.482 (0.412, 0.555)	0.227
Area (cm^2^)	3.725 (3.307, 4.109)	3.742 (2.969, 4.213)	0.929	3.507 (3.200, 4.126)	0.617
Grayscale	131.155 ± 14.141	130.676 ± 12.892	0.886	130.479 ± 16.17	0.806
AAP (°)	76.639 ± 8.876	75.642 ± 9.326	0.641	71.720 ± 11.775	0.025

*RPR* right puborectalis, *AAP* the angle between the arch tendineus levator ani and puborectalis

^†^Comparisons between the characteristics in the RPR of women with RPR pain and normal controls

^‡^Comparisons between the characteristics in the RPR of women with RIC pain and normal controls

### Comparison between women with IC pain and the control group

Women with LIC pain had a higher rate of deficiency in the LPR compared to the control group (29.6% vs. 5.6%, *P* < 0.001, Table 2). However, both the LIC pain group and the control group did not show any significant difference in focal thickening in the LPR. When comparing the deficiency rate and focal thickening rate of the RPR between women with RIC pain and the control group, no significant difference was observed (*P* > 0.001, Table 3). In terms of the length, minimum width, area, grayscale, and AAP of the LPR/RPR, the grayscale in LPR of women with LIC pain was found to be lower than that of the control group (median 121.786, IQR 112.712–128.940 vs. median 126.722, IQR 116.951–138.631, *P* < 0.05). Additionally, the right AAP of women with RIC pain was smaller than the control group (median 73.915, IQR 61.987–82.996 vs. median 77.033, IQR 70.082–83.336, *P* < 0.05). However, no correlation was found between the measurement indicators and the VAS score of LIC or RIC pain (*P* > 0.05).

### Comparison between the pain side and non-pain side in women with unilateral PR pain

A comparison was made between the PR on the pain side and the non-pain side in women with unilateral PR pain. The deficiency rate of the PR on the pain side was significantly higher than on the non-pain side (55.3% vs. 2.6%, *P* < 0.001). No focal thickening was found in these women (Table 4). The length, minimum width, area, grayscale, and AAP were compared between the PR on the pain side and the non-pain side. The grayscale of the PR on the pain side was higher than that on the non-pain side (median 128.858, IQR 117.777–134.395 vs. median 131.482, IQR 126.720–137.244, *P* < 0.001). However, no significant correlation was found between any of the measurement indicators and the VAS score of PR pain (*P* > 0.05).

**Table 4:** Observational and measured characteristics in the pain side/ non-pain side PR of women with unilateral PR or IC pain

	unilateral PR pain			unilateral IC pain		
Characteristic	Pain side (*n* = 38)	Non-pain side (*n* = 38)	*P* value	Pain side (*n* = 32)	Non-pain side (*n* = 32)	*P* value
Deficiency	21 (55.3%)	1 (2.6%)	< 0.001	4 (12.5%)	3 (9.4%)	1
Focal thickening	0	0	–	0	0	–
Length (cm)	5.361 ± 0.508	5.464 ± 0.513	0.120	5.496 ± 0.614	5.491 ± 0.480	0.951
Minimum width (cm)	0.418 (0.350, 0.529)	0.463 (0.390, 0.527)	0.375	0.463 ± 0.112	0.477 ± 0.108	0.577
Area (cm^2^)	3.717 ± 0.779	3.658 ± 0.659	0.379	3.686 ± 0.618	3.795 ± 0.715	0.120
Grayscale	126.369 ± 11.847	132.171 ± 12.427	< 0.001	128.367 (116.164, 137.199)	124.874 (118.422, 132.919)	0.139
AAP (°)	79.031 ± 10.305	77.900 ± 11.699	0.397	70.472 ± 11.801	76.077 ± 10.520	0.004

*PR* puborectalis, *IC* iliococcygeus, *AAP* the angle between the arch tendineus levator ani and puborectalis

### Comparison between the pain side and non-pain side in women with unilateral IC pain

In women with unilateral IC pain, there was no significant difference in the deficiency rate of the LPR/RPR between the pain side and the non-pain side. (*P* > 0.05). Additionally, no focal thickening was observed in the PR on either side (Table 4). The length, minimum width, area, grayscale, and AAP were compared between the PR on the pain side and that on the non-pain side. The AAP of the pain was found to be smaller than that of the non-pain side (median 69.108, IQR 62.313, 80.390 vs. median 73.894, IQR 68.252–83.686, *P* < 0.01). However, no significant correlation was found between any of the measurement indicators and the VAS score of the IC pain (*P* > 0.05).

### Multivariate analysis for PFMP

Based on the aforementioned results, it was observed that PR deficiency and focal thickening were the most prevalent characteristics in postpartum women with PFMP. There was a significant difference in the PR deficiency rate (39.0% vs. 7.0%, *P* < 0.001) and the focal thickening rate (11.7% vs. 0.7%, *P* < 0.001) between the PFMP group and the control group. Taking into account factors related to postpartum pelvic pain and dyspareunia from the baseline and previous literature, a multivariate logistic regression analysis of PFMP was conducted (Table 5). PR deficiency (AOR = 11.625, 95% CI = 4.557–29.658) and focal thickening (AOR = 16.891, 95% CI = 1.819–156.805) were associated with an increased risk of postpartum PFMP. On the other hand, lactation (AOR = 0.295, 95% CI = 0.091–0.951) was associated with a decreased risk of postpartum PFMP.

**Table 5:** Multivariable analysis of PFMP^a^

Variable	AOR (95% CI)	*P* value
Lactation	0.295 (0.091, 0.951)	0.041
PR deficiency	11.625 (4.557, 29.658)	< 0.001
PR focal thickening	16.891 (1.819, 156.805)	0.013

*AOR* Adjusted odds ratio, *95% CI* 95% confidence interval

_a_Adjusted with age, BMI, education, postpartum days, mode of delivery (cesarean or spontaneous), operative vaginal delivery, parity, birth weight, episiotomy, laceration, lactation and urinary incontinence

## Discussion

The current study utilized transvaginal ultrasound to evaluate the sonographic characteristics of the levator ani in postpartum women with PFMP. Several ultrasound changes, including PR deficiency, focal thickening, grayscale, and AAP, were found to be specific in patients with postpartum PFMP, making this study the first to establish a connection between ultrasound findings and PFMP, while also comparing them with normal controls.

Deficiency and focal thickening were frequently observed in the PR on the side with pain, and they were found to be significantly associated with postpartum PFMP. Previous studies have used various terms to describe levator muscle injuries, such as avulsion, defect, and deficiency. Dietz and Simpson(17)defined the avulsion of the levator muscle [17–19] as the “ discontinuity between the inferior pubic ramus and the puborectalis muscle” observed by transperineal ultrasound. The defect has been described in both transperineal and transvaginal ultrasound, namely “discontinuity between the levator ani muscle and the lateral wall of the pelvis,” which can be measured or estimated [20, 21]. The deficiency was first defined by Hudson [22], encompassing defects and avulsions, and can also be measured. In this study, the definition of “deficiency” included PR avulsion, defect, inflammation, edema, and microlesions that presented as hypoechoic. A retrospective cohort study used MRI to observe the levator ani muscle in 18 patients with postpartum pelvic pain lasting more than 6 weeks, among whom 33.3% had defects in their levator ani muscle [23]. In our results, 51.9% of the 77 patients with PFMP were found to have a deficiency in their sonographic images. Patients with PR or IC pain were more likely to have PR deficiency on the painful side compared to the same side in the control group. In patients with lateral PR or IC pain, the deficiency rate of their PR on the painful side was higher than that on the non-painful side. The characteristic of focal thickening is highly similar to the trigger point, which is a palpable and localized nodule with distinct pain upon compression. Although only 10 patients with PFMP were found to have focal thickening of the PR on the painful side, this characteristic was significantly associated with PR pain.

Interestingly, in our study, both deficiency and focal thickening in the patients with PFMP were observed to be close to the location of trigger points detected during palpation. Previous literature has described the ultrasonic manifestations of various skeletal myofascial pain trigger points as a spherical or elliptical hypoechoic lesion on B-mode, stiffness on elastography, and alterations in Doppler parameters such as high peak systolic velocity, low peak diastolic velocity, high blood volume, and high outflow resistance [9]. Some studies also suggested that trigger points may appear as hyperechoic “cotton ball” or linear structures on B-mode [24, 25]. In our study, the deficiency and focal thickening lesions were either hypoechoic or hyperechoic and exhibited a round or linear shape, which was consistent with previous literature. However, since we did not use any positioning tool to assist in identifying the exact location of the trigger point, further research is needed to determine if the observed deficiency or focal thickening lesions are indeed indicative of trigger points.

Given the inherent differences in muscle thickness and pelvic structure among different women, the measurement parameters comparing patients with PFMP and the control group were not entirely consistent between the left and right sides. To mitigate the effect of individual differences, we compared the parameters between the pain side and the non-pain side of the patients with unilateral pain. Among the patients with unilateral pain, we observed differences in the grayscale and AAP between the pain side and the non-pain side. Grayscale is a measurement index that quantifies muscle echogenicity, with higher values indicating higher muscle echogenicity [26]. In PFMP patients, hypertonicity of the levator ani muscles can lead to muscle hypoxia and accumulation of inflammatory factors, resulting in inflammatory changes in the levator ani muscles [21, 27]. Chronic muscle inflammation typically presents increasing echogenicity on ultrasound due to fibrous tissue replacement, while muscle inflammation less than 1 year usually presents relatively low echogenicity [28]. The women included in this study were postpartum for less than 1 year (65 ± 32 days). For women with unilateral PR pain, the grayscale of their PR on the pain side was less than that on the non-pain side (median 128.858, IQR 117.777–134.395 vs. median 131.482, IQR 126.720–137.244, *P* < 0.001), suggesting that PR in the pain side was relatively hypoechoic within 1 year after delivery. In addition to grayscale, we also measured AAP to indirectly detect the ultrasound changes in patients with unilateral IC pain. Yan et al. used MRI to observe the levator ani muscle of postpartum women and found lesions such as avulsion and micro-injury of the levator ani. However, due to the thin thickness and deep location of the IC, complete observation using ultrasound is not feasible. Therefore, we observed its beginning at the arch tendinous levator ani (ATLA). Spitznagle suggested that the pelvic floor myofascial pain and tension were related to the compensation mechanism triggered by the structural damage and functional needs of the adjacent muscle groups, as the levator ani is a wide-range muscle that maintains a constant tone to support the pelvic organs [27]. We hypothesized that the IC pain and spasm could be compensatory responses to levator ani injury, which may manifest as a tense state at the ATLA. Our results showed the AAP on the pain side was smaller than that on the non-pain side in patients with unilateral IC pain (median 69.108, IQR 62.313–80.390 vs. median 73.894, IQR 68.252–83.686, *P* < 0.001). This finding supports our hypothesis and the myofascial compensation theory, suggesting that AAP can be used as an ultrasound evaluation parameter for patients with IC pain.

In the multivariate analysis, our study revealed that lactation was associated with a lower risk of postpartum PFMP (AOR = 0.295, 95% CI = 0.091–0.951). This finding contrasts with previous studies that suggested lactation as a risk factor for postpartum pelvic pain [29, 30]. However, some studies have reported no independent association between lactation and postpartum pelvic pain and have even suggested lactation as a protective factor [31]. It is important to acknowledge that in our study, only the lactation status of the participants was considered, without collecting detailed information on lactation duration and reasons for stopping lactation. Therefore, further research that incorporates more comprehensive and detailed lactation information is warranted to better understand the complex relationship between lactation and PFMP.

### Limitation

The current study has several limitations that should be acknowledged. Firstly, there may have been inevitable differences in pelvic conditions among individuals, which could have resulted in inconsistencies in the comparisons between the left and right sides. However, by comparing the pain side and non-pain side in patients with unilateral pain, we attempted to minimize this bias. Secondly, this study was conducted at a single center with a small sample size, which may limit the generalizability of the findings. Further research with larger multi-center clinical trials is warranted to obtain more robust and reliable results. Thirdly, the ultrasound changes in pelvic floor muscle pain (PFMP) were observed before any treatments were administered. Future studies should consider comparing ultrasound findings before and after PFMP treatments to verify the effectiveness of the observed indicators and identify more meaningful indicators for clinical practice. Despite these limitations, our study provides valuable insights into the ultrasound evaluation of PFMP, and further research is warranted to build upon these findings and address the limitations.

## Conclusion

This study highlights the valuable information obtained from the sonographic evaluation of the levator ani muscle in postpartum women with pelvic floor muscle pain (PFMP). The ultrasound characteristics including PR deficiency, focal thickening, grayscale, and AAP, are specific to patients with postpartum PFMP. PR deficiency and focal thickening were frequently found in the pain muscle and closely associated with the location of trigger points identified during palpation. Further studies are needed to investigate whether these changes correspond to the trigger points and to elucidate the underlying pathophysiology of these changes in PFMP. These findings contribute to the understanding of PFMP and provide a foundation for future studies in this field.

### Supplementary Information

**Supplementary Material.**

## Data Availability

The data that support the findings of this study are available on request from the corresponding author. The date are not publicly available due to privacy or ethical restrictions.

## Abbreviations

PFMP: Pelvic floor myofascial pain
PR: Puborectalis
LPR: Left puborectalis
RPR: Right puborectalis
AAP: Arch tendinous levator ani and puborectalis
ICC: Intraclass correlation coefficient
AOR: Adjusted odds ratio
CI: Confidence interval
VAS: Visual Analogue Scale
ATLA: Arch tendineus levator ani
IC: Iliococcygeus
LIC: Left iliococcygeus
RIC: Right iliococcygeus
IQR: Interquartile range
