# Supplementary material for: Exploring transvaginal sonographic characteristics of the levator ani muscle in women with postpartum pelvic floor myofascial pain
Source: BMC Womens Health. 2024 Apr 18;24:245. doi: 10.1186/s12905-024-03052-9 (PMC11025161; doi:10.1186/s12905-024-03052-9)
Supplement: Supplementary file 1 — Supplementary Material. [file 12905_2024_3052_MOESM1_ESM.docx]

**Table A.1.** Interrater reliability of the measurement indications

| Measurement | ICC | 95% CI |
| --- | --- | --- |
| LPR-length | 0.924 | 0.740, 0.980 |
| LPR-width | 0.788 | 0.366, 0.943 |
| LPR-area | 0.759 | 0.324, 0.933 |
| LPR-grayscale | 0.885 | 0.598, 0.970 |
| LPR-deficiency length | 0.898 | 0.125, 0.993 |
| LPR-deficiency width | 0.944 | 0.320, 0.996 |
| LPR-deficiency area | 0.951 | 0.434, 0.997 |
| Left AAP | 0.947 | 0.737, 0.988 |
| RPR-length | 0.895 | 0.647, 0.972 |
| RPR-width | 0.925 | 0.744, 0.981 |
| RPR-area | 0.783 | 0.374, 0.940 |
| RPR-grayscale | 0.742 | 0.268, 0.928 |
| RPR-deficiency length | 0.916 | 0.339, 0.994 |
| RPR-deficiency width | 0.893 | 0.066, 0.993 |
| RPR-deficiency area | 0.938 | 0.288, 0.996 |
| Right AAP | 0.973 | 0.903, 0.993 |
| ICC, interclass correlation coefficient; 95% CI, 95% confidence interval;  LPR, left puborectalis; RPR, right puborectalis;  AAP, the angle between the tendinous arch of the levator anis and puborectalis. | | |

**Table A.2.** Interrater reliability of the observed indications

| Indication | Kappa value | 95% CI |
| --- | --- | --- |
| LPR-deficiency | 0.600 | 0.145, 1.000 |
| LPR-focal thickening | 0.783 | 0.389, 1.000 |
| RPR-deficiency | 0.755 | 0.308, 1.000 |
| RPR-focal thickening | 0.737 | 0.265, 1.000 |
| 95% CI, 95% confidence interval;  LPR left puborectalis; RPR, right puborectalis. | | |
